# Random Matrix Analysis of Ca^2+^ Signals in β-Cell Collectives

**DOI:** 10.3389/fphys.2019.01194

**Published:** 2019-09-18

**Authors:** Dean Korošak, Marjan Slak Rupnik

**Affiliations:** ^1^Faculty of Medicine, Institute for Physiology, University of Maribor, Maribor, Slovenia; ^2^Faculty of Civil Engineering, Transportation Engineering and Architecture, University of Maribor, Maribor, Slovenia; ^3^Center for Physiology and Pharmacology, Medical University of Vienna, Vienna, Austria; ^4^Alma Mater Europaea - European Center Maribor, Maribor, Slovenia

**Keywords:** collective sensing, pancreatic islets, random matrix theory (RMT), metabolic code, Ca^2+^ imaging, Ca^2+^ signaling, correlations, intercellular communication

## Abstract

Even within small organs like pancreatic islets, different endocrine cell types and subtypes form a heterogeneous collective to sense the chemical composition of the extracellular solution and compute an adequate hormonal output. Erroneous cellular processing and hormonal output due to challenged heterogeneity result in various disorders with diabetes mellitus as a flagship metabolic disease. Here we attempt to address the aforementioned functional heterogeneity with comparing pairwise cell-cell cross-correlations obtained from simultaneous measurements of cytosolic calcium responses in hundreds of islet cells in an optical plane to statistical properties of correlations predicted by the random matrix theory (RMT). We find that the bulk of the empirical eigenvalue spectrum is almost completely described by RMT prediction, however, the deviating eigenvalues that exist below and above RMT spectral edges suggest that there are local and extended modes driving the correlations. We also show that empirical nearest neighbor spacing of eigenvalues follows universal RMT properties regardless of glucose stimulation, but that number variance displays clear separation from RMT prediction and can differentiate between empirical spectra obtained under non-stimulated and stimulated conditions. We suggest that RMT approach provides a sensitive tool to assess the functional cell heterogeneity and its effects on the spatio-temporal dynamics of a collective of beta cells in pancreatic islets in physiological resting and stimulatory conditions, beyond the current limitations of molecular and cellular biology.

## Introduction

Pancreatic islets are collectives of endocrine cells. Based on the end hormone that these cells exocytose in a Ca^2+^-dependent manner after being stimulated, several types of cells have been described to compose an islet, with 3 dominant cell types: alpha, beta and delta (Briant et al., 2017). Islets from different parts of pancreas can contain different fractions of each of these cell types, but the bulk cellular mass in a typical islet is in an non-diabetic organism composed of a collectives of insulin secreting beta cells (Dolenšek et al., 2015; Rorsman and Ashcroft, 2017). Early studies assumed these beta cell collectives to be a rather homogeneous population of cells, however, the subsequent functional analyses have revealed a remarkable degree of heterogeneity even in dissociated beta cells in culture. The beta cells were found to differ in a number of physiological parameters, among others in glucose sensitivity and Ca^2+^ oscillation pattern (Zhang et al., 2003), electrical properties (Misler et al., 1986), redox states (Kiekens et al., 1992), or pattern on cAMP oscillations (Dyachok et al., 2006). These early quests (Pipeleers, 1992) have been mostly searching for morphological, physiological and molecular features that would presumably satisfy at least 3 criteria: (a) entitle special roles for individual cells within the collectives, (b) remain valid even after cell dissociation, and (c) enable to trace embryonic and postnatal development as well as changes during pathogeneses of different forms of diabetes. Recent onset of efficient high-throughput analyses has catapulted these approaches on mostly dissociated cells to a completely new level and enabled identification of a multitude of functional and non-functional subpopulations with their functional characteristics, gene and protein expression, incidences and diabetes-related changes (for a recent review refer to Benninger and Hodson, 2018). A major limitation of these analyses is that the subpopulations described in different studies have relatively little in common and currently their translational relevance is weak. This is, however, not surprising since these approaches primarily deal with sample averages and present merely a small number of discrete snapshots of a very dynamic complex activity. Nevertheless, they turned out to be extremely useful in initial attempts to construct a pseudotime map of the sequence of events in pancreatic endocrine system (Damond et al., 2019) and its interaction with the immune system (Wang et al., 2019) during the progression of type 1 diabetes mellitus (T1D) in humans. Still, better tools are needed to assess the general rules underlying functional heterogeneity in a real-time spatio-temporal dynamics within collectives of beta cells or collectives of any other cell types in an organism. Instead of an ultra-reductionist approach to precisely associate individual molecular markers yielding more or less random correlations in respect to functional heterogeneity, a network of cells is first segmented to their level of functional influence expressed in high deviating eigenvalues to lead a more efficient further discovery of few collective parameters which may also have an identifiable molecular signature. Pancreatic islets have been described as broad scale network (Stožer et al., 2013b) and the search for important principal components is well justified.

The fact is that most of what we know about pancreatic beta cells has been gained by studying dissociated beta cells in cell culture. Therefore, even phenomena that can only be observed in isolated groups of electrically coupled beta cells, like electrical activity (Rorsman and Trube, 1986) or cytosolic Ca^2+^ oscillation, are currently still mostly modeled within the framework of a single cell excitability (Sherman et al., 1988; Bertram et al., 2018). However, each beta cell interacts with several immediate and distal neighboring cells in a pancreatic islet, implicating high-ordered interactions between a large number of elements. Therefore, there is a rich exchange of signals within such a beta cell collective, both through direct cell-cell coupling (Bavamian et al., 2007) as well as through paracrine signaling (Caicedo, 2013; Capozzi et al., 2019). Such an organization necessarily yields a complex response patterns of cell activity after stimulation with physiological or pharmacological stimuli. Until recently, the richness of the aforementioned cell-cell interactions also could not be experimentally documented. However, recent technological advancements made it possible to use various optical tools to address these issues (Frank et al., 2018). For example, the functional multicellular imaging (fMCI) enabled completely new insights into our understanding of a beta cell in an islet as a biological network (Dolenšek et al., 2013; Stožer et al., 2013a,b). The dynamics of a measurable physiological parameter can namely be recorded in hundreds of beta cells within their intact environment (Speier and Rupnik, 2003; Marciniak et al., 2014) simultaneously. The measured oscillatory cytosolic Ca^2+^ concentration changes, which are required to drive insulin release turned out to be a practical tool to trace cellular activity and fundamental to study their interactions in such big collectives (Dolenšek et al., 2013; Stožer et al., 2013a,b). With the use of the tools of statistical physics we and others reconstructed, for example the complex network topologies in beta cell activation, activity and deactivation during transient glucose challenges (Stožer et al., 2013b; Gosak et al., 2015; Markovič et al., 2015; Johnston et al., 2016). As in some other, previously analyzed biological systems, also for the pancreatic islets, the minimal model incorporating pairwise interactions provides accurate predictions about the collective effects (Schneidman et al., 2006; Korošak and Slak Rupnik, 2018).

Along these lines we have recently shown that beta cell collectives work as a broad-scale complex networks (Stožer et al., 2013b; Markovič et al., 2015; Gosak et al., 2017a), sharing similarities in global statistical features and structural design principles with internet and social networks (Milo et al., 2002; Barabási and Márton Pósfai, 2016; Daniels et al., 2016; Perc et al., 2017; Duh et al., 2018). In addition to complex network description when strong cell-cell interaction are primarily taken into account, the analyses of weak pairwise interaction enabled us to use a spin glass model (Korošak and Slak Rupnik, 2018), as well as the assessment of self organized criticality (Bak, 2013; Marković and Gros, 2014; Gosak et al., 2017b; Stožer et al., 2019), also often found in biological samples (Schneidman et al., 2006). The important result from these functional studies is that a faulty pattern of hormone release due to deviating numbers of individual cell types or changes in their function lead to one of the forms of a large family of metabolic diseases called diabetes mellitus (American Diabetes Association, 2014; Pipeleers et al., 2017; Skelin Klemen et al., 2017; Nasteska and Hodson, 2018; Capozzi et al., 2019).

The basic object that we study here is the correlation matrix **C** with elements computed from measured Ca^2+^ signals:

(1) $$\begin{array}{l} \mathrm{Corr}\left(y_{i},y_{j}\right)=C_{ij}=\frac{\langle y_{i}y_{j}\rangle -\langle y_{i}\rangle \langle y_{j}\rangle }{\sigma_{i}\sigma_{j}}, \end{array}$$

where *y*_*i*_(*t*) is the i-th time series of Ca^2+^ signal out of *N* signals measured simultaneously in a collective of pancreatic beta cells.

Random matrix theory (Guhr et al., 1998; Mehta, 2004) (RMT) is concerned with statistical properties of matrices with random elements. Applying RMT to correlation matrices, we study the spectrum of the correlation matrix **C** given by the set of its eigenvalues λ_*n*_:

(2) $$\begin{array}{l} \text{C}{\text{u}}_{n}=\lambda_{n}{\text{u}}_{n}, \end{array}$$

where **u**_*n*_ are the corresponding eigenvectors.

Statistical properties of the spectra of random correlation matrices for *N* uncorrelated time series with *M* random elements where *q* = *N*/*M* is finite in the limit *N, M* → ∞ are known analytically (Marchenko and Pastur, 1967; Bun et al., 2017). The eigenvalue probability density is:

(3) $$\begin{array}{l} \rho \left(\lambda \right)=\frac{1}{2\pi q\lambda }\sqrt{\left(\lambda_{+}-\lambda \right)\left(\lambda -\lambda_{-}\right)}, \end{array}$$

where the spectral boundaries are:

(4) $$\begin{array}{l} \lambda_{\pm }={\left(\sqrt{q}\pm 1\right)}^{2} \end{array}$$

When the spectrum of the correlation matrix is unfolded (Guhr et al., 1998) by mapping eigenvalues λ_*k*_ → ξ_*k*_ so that the probability density of the unfolded eigenvalues is constant ρ(ξ) = 1, the RMT predicts that the distribution *P*(*s*) of nearest neighbor spacings *s*_*k*_ = ξ_*k*+1_ − ξ_*k*_ is approximately given by the Wigner surmise (Mehta, 2004):

(5) $$\begin{array}{l} P\left(s\right)=\frac{\pi }{2}s\exp\left(-\frac{\pi }{4}s^{2}\right). \end{array}$$

Possible pair correlations in the eigenvalue spectrum on scales larger than nearest neighbors can be revealed with the use of variance of *n*_ξ_(*L*), the number of eigenvalues in the interval of length *L* around eigenvalue ξ. This number variance (Mehta, 2004) is given by:

(6) $$\begin{array}{l} \Sigma^{2}\left(L\right)=\langle {\left(n_{\xi }\left(L\right)-L\right)}^{2}\rangle . \end{array}$$

If the eigenvalue spectrum is poissonian the number variance is Σ^2^(*L*) ~ *L*, while real, symmetric random matrices exhibit correlated spectra for which RMT predicts Σ^2^(*L*) ~ log*L* (Mehta, 2004).

Previous work using RMT in different systems, e.g., on statistics of energy levels of complex quantum systems (Guhr et al., 1998; Mehta, 2004) or correlations in financial markets (Plerou et al., 2002) identified that a bulk of the eigenvalue spectrum agreed with RMT predictions, which suggested a large degree of randomness in the measured cross-correlations in these systems. Only a small fraction of typically a few percent of eigenvalues were found to deviate from universal RMT predictions and were instrumental to identify system specific, non-random properties of the observed systems and yielding key information about the underlying interactions. Biological systems are often complex with large number of interacting parts, high dimensional systems that are basically black boxes “in which a large number of particles are interacting according to unknown laws” (Dyson, 1962). One way to approach such high dimensional systems is to look at the spectrum of the covariance matrix and try to find few principal components that describer most of system variance. This method, principal component analysis (PCA), has been suggested as “‘hypothesis generating’ tool creating a statistical mechanics frame for biological systems modeling” (Giuliani, 2017). PCA works best for systems where we can find few eigenvalues in the covariance spectrum well separated from the bulk and the system can be described in low dimensional space. Usually, there is no clear separation in the eigenvalue spectrum and other methods such as RMT or methods using renormalization group approach (Bradde and Bialek, 2017) are more suitable. In biological systems, RMT has been used to filter out critical correlations from data-rich samples in genomic, proteomic and metabolomic analyses (Luo et al., 2006; Agrawal et al., 2014), as well as in brain activity measured by EEG (Šeba, 2003) and dynamic brain networks constructed from fMRI data (Wang et al., 2015, 2016). While eigenvalue spacing distributions showed agreement with RMT predictions, the number variance distributions often display deviations pointing to the physiologically relevant reduction in correlated eigenvalues fluctuations with partially decoupled components transiting toward Poisson distribution (Šeba, 2003). Such transitions have also been used as an objective approach for the identification of functional modules within large complex biological networks (Luo et al., 2006). Additionally, as for protein-protein interactions in different species, these latter deviation from RMT predictions has been interpreted as an evidence to support the prevalence of non-random mutations in biological systems (Agrawal et al., 2014).

In this paper we used the RMT approach to test the cross-correlations in the cytosolic Ca^2+^ oscillations under non-stimulated and glucose stimulated conditions. We demonstrate that statistical properties of cross-correlations based on functional multicellular imaging data follows those predicted by RMT, with both high- and low-end deviating eigenvalues, suggesting local as well as global modes driving this correlation in functional islet. In addition, our results show that the long range correlations in eigenvalue spectrum deviate in a stimulus dependent manner.

## Dataset Description

We define beta cell collective activity to sense nutrients and produce metabolic code as the relevant constraining context for the physical outcomes of analysis (Ellis and Kopel, 2018; Korošak and Slak Rupnik, 2018). Our data consist of a typical Ca^2+^ activity recorded by multicellular imaging on an islet of Langerhans of fresh mouse pancreatic slice. All data analysis has been performed using custom scripts in Python 3.5 software and customized scripts (RMThreshold) in R software. We used raw data for each calcium signal, but we detrended the signals to remove possible sources of spurious correlations due to systematic slow variations caused by the imaging technique. A common problem in the analysis of fMCI Ca^2+^ signals in living tissue is selection of regions of interest corresponding to a true signal originating from a cytosolic area of an individual cell and not two or more neighboring cells. In practice the reproducibility of the results depends on the level of experience of the operator to subjectively recognize structure from the patterns of activity. While we are primarily interested in the activity of a large population of cells, their interactions/correlations and their collective response, it is crucial that this signals originate from regions of interest that correspond to individual cells. Collectives of cells, like beta cells in the islet of Langerhans are densely packed structures, where extracellular space and the cell membrane represent a relatively small to negligible cross-section area on the image of two-dimensional optical section obtained by confocal microscopy. Therefore we decided to avoid the aforementioned subjectivity issue by the random sampling of pixel level signals in the recorded time series. For this analysis we randomly selected *N* = 4000 signals out of the complete dataset of 256 × 256 signals each *M* = 23,820 timesteps long (about 40 min recordings at 10 Hz resolution). Glucose concentration was changed during the recording from 6 mM (lasting about first 5,000 timesteps) to 8 mM and back to 6 mM (approx. last 5,000 timesteps) near the end of experiment.

The source of correlations in a cell population where the terminal action is a calcium-dependent process (e.g., exocytosis of insulin in beta cells) are the individual events in a form of plasma membrane ion channel or transporter activity, internal membrane ion channel or transporter activity, as well as calcium leak from activated immediate neighboring beta cell (Berridge et al., 2000). The correlations between the activities of beta cells depend strongly upon the glucose concentration (Dolenšek et al., 2013; Markovič et al., 2015), however in the physiological plasma glucose range (6–9 mM), most correlations are weak (Korošak and Slak Rupnik, 2018), so that the probability of detecting co-activation basically equals the product of the probabilities of activities of individual beta cells. The correlations are statistically significant for almost all pairs of immediate neighbors.

## Results

**Figure 1:**
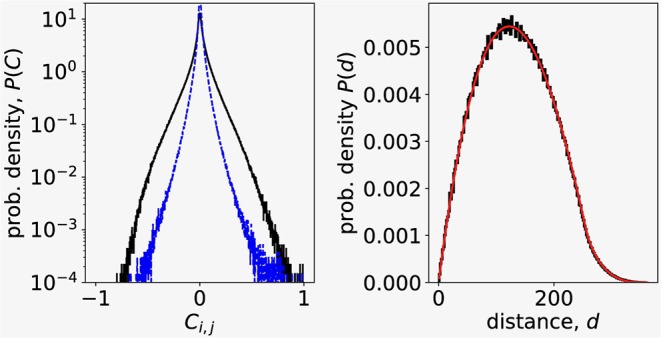
**(Left)** Correlation coefficient distribution of *N* = 4000 Ca^2+^ signals randomly chosen from experimental dataset (outer, black line). Dashed blue line is the distribution of correlations between residuals after removing the influence of the largest eigenvalue in signals. **(Right)** Distribution of distances between signals randomly chosen from Ca^2+^ imaging data. Black line is the experimental data, thinner red line is the theoretical distribution of distances between random points in a square (Philip, 2007).

The distribution of correlation coefficients reveals that most of the correlations between the pairs of Ca^2+^ signals are weak, but there is also non-negligible contribution of highly correlated pairs of signals (Figure 1, left, black outer line). We also checked the sampling procedure by comparing the computed distribution of distances between pairs of randomly chosen points from 256 × 256 image square to the analytical probability distribution of distances between two random points in a square (Philip, 2007) (Figure 1, right). We found a perfect match between the distance distribution computed from data and the theoretical distribution, confirming that our random sampling of data points was non-biased.

**Figure 2:**
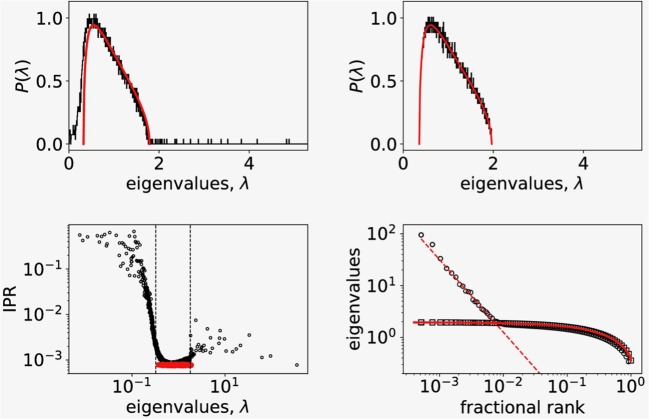
**(Top left)** Probability distribution of eigenvalues of the empirical correlation matrix for *N* = 4,000 randomly picked signals (black solid line) compared to the distribution of eigenvalues of random correlation matrix of the same size (red solid line). **(Top right)** Probability distribution of eigenvalues of the surrogate correlation matrix constructed from shuffled empirical values (black solid line) compared to the random correlation matrix of the same size (red solid line). **(Bottom left)** Inverse participation ratio plot of eigenvalues showing a random band matrix structure of C with large IPR values at both edges of the eigenvalue spectrum. The dashed vertical lines show RMT bounds. The IPR spectrum for randomized correlations is shown in red. **(Bottom right)** The fractional rank plot of the entire spectrum of eigenvalues (open dots). For comparison we added the same plot of eigenvalues of correlation matrix computed from randomized data (open squares). The full line shows the fractional rank plot of eigenvalue spectra obtained from distribution given by Equation (3). The shape of the distribution of large eigenvalues points to a scaling relationship.

Guided by the observed non-gaussian nature of correlation distribution (Figure 1, left) we explored a detailed structure of the correlation matrix, since distribution of correlation coefficients only partially hints to the nature of cell to cell coordination. To this end we computed the eigenvalues and eigenvectors of the correlation matrix (Equation 2) and compared the obtained eigenspectrum with the RMT prediction. In Figure 2 (top left) we show the distribution of eigenvalues that belong to the empirical correlation matrix (black trace) and the RMT prediction (red line) given by Equation (3). While most of the eigenvalues falls within the limits λ_±_ of the RMT spectrum, there are also significant deviations from RMT prediction. We found the largest empirical eigenvalue λ_*max*_ two orders of magnitude away from the upper limit of the RMT spectrum, and also a part of the empirical spectrum that extends below the lower RMT limit. To see if the deviations from the RMT are inherent to the measured Ca^2+^ signals, we prepared a surrogate dataset by randomly shuffling each signal's time series. We then computed the correlation matrix and its eigenvalue spectrum from randomized surrogate dataset. As shown in Figure 2 (top right), the match between the eigenvalue distribution of randomized dataset and RMT is perfect.

Previous RMT analysis of stock correlations in financial markets consistently showed (Laloux et al., 1999; Plerou et al., 1999, 2002) that the distribution of components of the eigenvector **u**_*max*_ corresponding to largest eigenvalue λ_*max*_ strongly deviates from Gaussian form, suggesting that this mode reflects the collective response of the system to the stimuli. In our case this corresponds to collective response of beta-cells to glucose stimulus. In a linear statistical model for Ca^2+^ signals, we model the response common to all beta-cell with *Y*(*t*) and the signals are expressed as:

(7) $$\begin{array}{l} y_{i}\left(t\right)=a_{i}+b_{i}Y\left(t\right)+\delta y_{i}\left(t\right), \end{array}$$

where δ*y*_*i*_(*t*) is the residual part of each signal. Coefficients *a*_*i*_, *b*_*i*_ are obtained by regression. Following Plerou et al. (2002) we approximated the common response *Y*(*t*) with the projection of all signals on the largest eigenvector:

(8) $$\begin{array}{l} y_{max}\left(t\right)=\overset{N}{\underset{i=1}{\sum }}u_{i}\left(\lambda_{max}\right)y_{i}\left(t\right), \end{array}$$

where *u*_*max, i*_ is the i-th component of the eigenvector corresponding to largest eigenvalue λ_*max*_. To see the influence of the collective response to the distribution of correlation coefficents, we computed using *Y* = *y*_*max*_ the residuals δ*y*_*i*_(*t*) for all *N* signals and their correlations *C*_*res*(*i, j*)_ = Corr(δ*y*_*i*_, δ*y*_*j*_). The dashed blue line (inner trace) on Figure 1 (left) shown the distribution of **C**_*res*_ and reveals that the collective response predominantly contributes to large correlations.

To test further if the largest eigenvalue and the corresponding eigenvector capture the collective calcium response we compared the average signal $\bar{y}\left(t\right)=1/N\underset{j}{\sum }y_{j}\left(t\right)$ with *y*_*max*_. The correlation between signals projected on the largest eigenvalue mode and mean signal was high: $Corr\left(y_{max},\bar{y}\right)\approx 0.8$, confirming the expectation that the largest eigenvalue represents collective effect. Similarly, we checked how similar are the signals corresponding to the bulk RMT eigenvalues:

(9) $$\begin{array}{l} y_{bulk,i}\left(t\right)=\overset{N}{\underset{j=1}{\sum }}u_{j}\left(\lambda_{i}\right)y_{j}\left(t\right), \end{array}$$

where λ_*i*_ is the eigenvalues from the RMT interval [λ_+_, λ_−_]. The computed correlation between signals projected on bulk eigenvectors and mean signal, averaged over all signals was $<\mathrm{Corr}\left(y_{bulk,i},\bar{y}\right)>=-0.0044\pm 0.0047$ suggesting no correlation between the mean signal and signals coming from the bulk RMT regime. To further characterize the eigenvector structure of the empirical Ca^2+^ correlation matrix, we looked at the inverse participation ratio (IPR) of eigenvector **u**(λ) corresponding to eigenvalue λ defined as (Plerou et al., 1999, 2002):

(10) $$\begin{array}{l} I\left(\lambda \right)=\overset{N}{\underset{j}{\sum }}{\left(u_{j}\left(\lambda \right)\right)}^{4}. \end{array}$$

The value of 1/*I*(λ) reflects the number of nonzero eigenvector components: if an eigenvector consist of equal components $u{\left(\lambda \right)}_{i}=1/\sqrt{\left(N\right)}$ then 1/*I*(λ) = *N*, in other extreme case 1/*I*(λ) = 1 when an eigenvector has one component equal to 1 and all others are zero.

Figure 2 (lower left) shows the computed values of IPR for all eigenvectors as function of corresponding eigenvalue. The red datapoints are the IPR data computed for the surrogate, randomized timeseries data for which we found 1/*I* ~ *N* as expected. We found similarly values 1/*I* ~ *N* for the largest eigenvalues of the empirical spectrum (black datapoints) suggesting that to this eigenvectors almost all signals contribute. Deviations from flat RMT prediction at the edges of the RMT spectrum ([λ_+_, λ_−_] interval, vertical dashed lines) with large *I*(λ) values suggests that these states are localized with only a few signals contributing. This points to a complex structure of the empirical correlation matrix **C** with coexisting extended and localized eigenvectors similar to one found in correlations in financial markets (Plerou et al., 1999, 2002). In addition, as shown in Figure 2 (lower right, open dots), we observe a scaling behavior in rank-ordered plot of eigenvalues of empirical correlation matrix that has been connected with a fixed point in renormalization group sense (Bradde and Bialek, 2017; Meshulam et al., 2018). For comparison, we plot also the rank-ordered eigenvalues of randomized data (open squares) and RMT prediction based on eigenvalue density given by Equation (3) (full line) which perfectly describes the randomized dataset. The observed scaling of eigenvalues hints toward the critical behavior that was conjectured for beta-cell collective at the transition from glucose non-stimulated to stimulated phase (from 6 mM to 8 mM) (Gosak et al., 2017b; Stožer et al., 2019).

**Figure 3:**
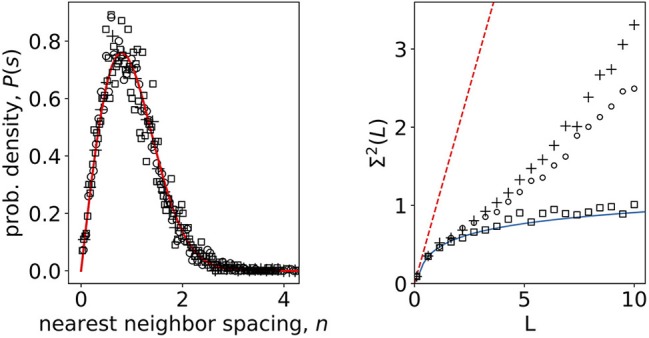
**(Left)** Nearest-neighbor spacing distribution of empirical correlation matrix eigenvalues of calcium signals in non-stimulatory and stimulatory regime. Open squares: shuffled, randomized data; open dots: 8 mM glucose; crosses 6 mM glucose; full line Wigner surmise (Equation 5). **(Right)** Number variance of eigenvalue spectra of calcium signals. Open squares: shuffled, randomized data; open dots: 8 mM glucose; crosses 6 mM glucose; full line RMT prediction *Σ*^2^(*L*) = 1/π^2^(log(2πL) + 1 + γ – π^2^/8) (Mehta, 2004); dashed line Poissonian limit *Σ*^2^(*L*) = *L*.

To explore the statistical differences of signals in non-stimulated and stimulated phase, we separated the original data into two groups of *N* signals each with *M* = 10^4^ timesteps corresponding to response to 6 and 8 mM glucose stimuli. For each group we computed the unfolded eigenvalue spectra and also for randomized data. The results for the nearest-neighbor spacing and number variances are shown in Figure 3. For nearest-neighbor spacing distribution we find a good agreement with the RMT prediction both, for non-stimulatory and stimulatory conditions, as well as shuffled stimulated data. All three datasets are well described with the Wigner surmise (Equation 5), so nearest-neighbor spacing does not seem to be sensitive to stimuli changes. On the other hand, however, the number variance is sensitive to stimuli change already during physiological stimulation of the beta-cell collective. The random matrix prediction is in this case valid for shuffled stimulated data only (Figure 3, right).

## Discussion

The unique spatio-temporal resolution of functional multicellular imaging and sensitivity of advanced statistical approaches for a plethora of different modes of complex network scenarios and levels of criticality, makes these approaches a method of choice to assess the nature of cell-cell interactions under different stimulation conditions. At the same time it enables us to test the validity of experimental designs for study of beta cell function, primarily in the domain of stimulation strength and dynamics. We suggest that without such validation the most critical events in the activation chain within the beta cell collective have been and shall be overlooked or misinterpreted (Stožer et al., 2019). The predominant use of supraphysiological glucose concentration can namely severely deform the relatively slow beta cell recruitment in a collective at physiological glucose concentrations (Stožer et al., 2013a; Gosak et al., 2017a) and miss the typically segregated network clusters of Ca^2+^ events (Benninger and Piston, 2014; Markovič et al., 2015; Westacott et al., 2017), turning critical behavior into disruptive supracritical activity (Gosak et al., 2017b; Stožer et al., 2019). Only under rather narrow physiological conditions it shall be possible to extract the fine structure of cell-cell interactions causing long-term and efficient cell collaboration with the collective. Breaking apart this delicate structure of cell-cell interaction does result in a massive activity, which can be readily described by tools of statistical physics, but this activity does not necessarily serve its physiological or biological purpose (Ellis and Kopel, 2018).

A common denominator of the previous attempts to categorize different beta cell types points to some metabolic and secretory features that can be either reproduced between different classifications or not. Usually there exist a bulk of one subtype and one or more less frequent subtypes (Benninger and Hodson, 2018). These less frequent subtypes can nevertheless have important regulatory roles that may not be immediately apparent. This issue is particularly critical if the frequency of a beta cell subtype represents only a couple of percent of the entire beta cell population in an islet. Along these lines there have been some indications regarding the beta cell subtypes that can serve as pacemakers or hubs within a dynamic islet cell network (Johnston et al., 2016; Lei et al., 2018), however due to the nature of complexity of network features, we may still be short of evidence for definitive conclusions. The full description of heterogeneity of endocrine cells within an islet, ultimately producing a adequate release of hormones is therefore still lacking. In trying to grasp this complexity, it is important to take into account interaction of beta cell collectives with other cell types in and around an islet, like glucagon-secreting alpha cells (Svendsen et al., 2018; Capozzi et al., 2019) or somatostatin secreting delta cells (Rorsman and Huising, 2018) as well as neurons and glial cells (Meneghel-Rozzo et al., 2004), but also endothelial, immune cells (Damond et al., 2019), as well as acinar and ductal cells (Bertelli and Bendayan, 2005).

Random matrix theory is a fitting mathematical framework which provides powerful analytical tools to separate cell-cell interactions happening by chance from those produced by specific coordinated interactions after a changed chemical composition of cell's surrounding. In the financial sector, adequate asset allocation and portfolio risk-estimation can lead to a higher profit and is therefore clear why it makes sense to invest time into cross-correlation analyses (Plerou et al., 2002). But what would be the gain of knowing that randomness of cell-cell correlation matrices is physiologically regulated? Firstly, we suggest that the analysis of the universal properties of empirical cross-correlations is a valuable tool to identify distinct types and further subtypes of endocrine cells within an islet through their non-local and local effects. The largest eigenvalue of **C** namely represents the influence of non-local modes common to all measured Ca^2+^ fluctuations. Other large eigenvalues can be used to address cross-correlations between cells of the same type, cells with specific functions in the collective or that these cells reside in topologically similar area of the islet. Quantifying correlations between different beta cells in an islet is therefore an exciting scientific effort that can help us understand cell communities as a complex dynamical system, estimate the amount of factors ruling the system or potential presence of a stress situation (Gorban et al., 2010).

The large values of inverse participation ratio (IPR) (Figure 2, bottom left) compared to the IPR values in the bulk, indicate that only a few cells contribute to these eigenstates with eigenvalues at the edges of the RMT bulk spectrum. In contrast, all cells contribute to the eigenstates corresponding to the largest eigenvalues. This means that we find delocalized states for the largest eigenvalues and localized states as we move toward the RMT edge of the spectrum. Similar findings were recently reported in RMT analysis of single-cell sequencing data (Aparicio et al., 2018), where the spectrum of covariance matrix of single-cell genomic data followed RMT predictions with deviations at the bulk edge. The localized states at the edge of the bulk spectrum were connected with the true biological signal.

Our results show that the number variance reflecting the correlation between subsequent eigenvalues (a measure for long range correlations in eigenvalue spectrum) follows the RMT predictions up to a certain distance *L*, however at larger distances it starts to deviate in a stimulus dependent manner, suggesting structural features in the beta cell network. Transitions between Poissonian and GOE statistics in biological systems have been previously described during the process of either integration or segregation of complex biological networks, showing various degrees of long range correlations at various physiological conditions (Luo et al., 2006). This understanding has a vital practical value since it can help decipher different roles that beta cells can play in a collective and to further validate the importance, if any, of previously defined and continuously appearing novel molecular markers of beta cell heterogeneity (Benninger and Hodson, 2018; Damond et al., 2019; Wang et al., 2019). An advanced knowledge about the dynamic properties of the functional cell types will shed a new light into understanding of physiological regulation of insulin release and the assessment of perils of stimulation outside of the physiological range. Furthermore, it can help us elucidate the mechanisms on how this function changes during the pathogenesis of different forms of diabetes mellitus and lead us to novel approaches of therapy planning and prevention. And finally, it can help us understand the general principles ruling the interactions in collectives of other cell types.

## Data Availability Statement

The raw data used in this research is available upon request to the corresponding author.

## Author Contributions

All authors listed have made a substantial, direct and intellectual contribution to the work, and approved it for publication.

### Conflict of Interest

The authors declare that the research was conducted in the absence of any commercial or financial relationships that could be construed as a potential conflict of interest.
