# Corrigendum: Random Matrix Analysis of Ca^2+^ Signals in β-Cell Collectives

**DOI:** 10.3389/fphys.2019.01322

**Published:** 2019-10-16

**Authors:** Dean Korošak, Marjan Slak Rupnik

**Affiliations:** ^1^Faculty of Medicine, Institute for Physiology, University of Maribor, Maribor, Slovenia; ^2^Faculty of Civil Engineering, Transportation Engineering and Architecture, University of Maribor, Maribor, Slovenia; ^3^Center for Physiology and Pharmacology, Medical University of Vienna, Vienna, Austria; ^4^Alma Mater Europaea - European Center Maribor, Maribor, Slovenia

**Keywords:** collective sensing, pancreatic islets, random matrix theory (RMT), metabolic code, Ca^2+^ imaging, Ca^2+^ signaling, correlations, intercellular communication

In the original article, there was a mistake in Figure 3 as published. Despite careful examination of the text and references in the proof, we unfortunately failed to notice that the incorrect Figure 3 was used. The correct Figure 3 appears below.

**Figure 3:**
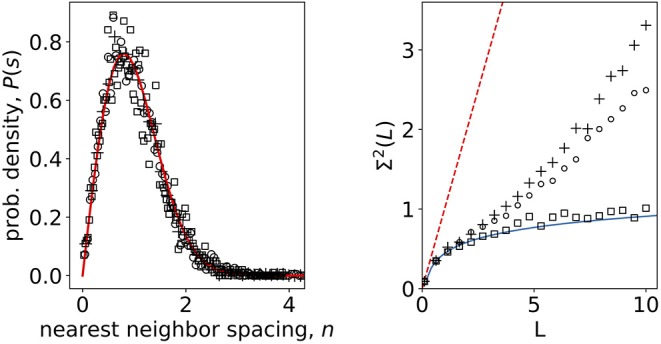
**(Left)** Nearest-neighbor spacing distribution of empirical correlation matrix eigenvalues of calcium signals in non-stimulatory and stimulatory regime. Open squares: shuffled, randomized data; open dots: 8 mM glucose; crosses 6 mM glucose; full line Wigner surmise (Equation 5). **(Right)** Number variance of eigenvalue spectra of calcium signals. Open squares: shuffled, randomized data; open dots: 8 mM glucose; crosses 6 mM glucose; full line RMT prediction *Σ*^2^(*L*) = 1/π^2^(log(2πL) + 1 + γ – π^2^/8) (Mehta, 2004); dashed line Poissonian limit *Σ*^2^(*L*) = *L*.

The authors apologize for this omission and state that this does not change the scientific conclusions of the article in any way. The original article has been updated.

